# Prediction of Undrained Shear Strength by the GMDH-Type Neural Network Using SPT-Value and Soil Physical Properties

**DOI:** 10.3390/ma15186385

**Published:** 2022-09-14

**Authors:** Mintae Kim, Osman Okuyucu, Ertuğrul Ordu, Seyma Ordu, Özkan Arslan, Junyoung Ko

**Affiliations:** 1 School of Civil, Environmental, and Architectural Engineering, Korea University, Seoul 02841, Korea; 2 Department of Civil Engineering, Tekirdağ Namık Kemal University, Tekirdağ 59860, Turkey; 3 Department of Environmental Engineering, Tekirdağ Namık Kemal University, Tekirdağ 59860, Turkey; 4 Department of Electronics and Communication Engineering, Tekirdağ Namık Kemal University, Tekirdağ 59860, Turkey; 5 Department of Civil Engineering, Chungnam National University, Daejeon 34134, Korea

**Keywords:** undrained shear strength, standard penetration test, group method of data handling, random forest, support vector regression, extreme gradient boosting

## Abstract

This study presents a novel method for predicting the undrained shear strength (*c_u_*) using artificial intelligence technology. The *c_u_* value is critical in geotechnical applications and difficult to directly determine without laboratory tests. The group method of data handling (GMDH)-type neural network (NN) was utilized for the prediction of *c_u_*. The GMDH-type NN models were designed with various combinations of input parameters. In the prediction, the effective stress (*σ_v_*’), standard penetration test result (*N_SPT_*), liquid limit (*LL*), plastic limit (*PL*), and plasticity index (*PI*) were used as input parameters in the design of the prediction models. In addition, the GMDH-type NN models were compared with the most commonly used method (i.e., linear regression) and other regression models such as random forest (RF) and support vector regression (SVR) models as comparative methods. In order to evaluate each model, the correlation coefficient (R^2^), mean absolute error (MAE), and root mean square error (RMSE) were calculated for different input parameter combinations. The most effective model, the GMDH-type NN with input parameters (e.g., *σ_v_*’, *N_SPT_*, *LL, PL, PI*), had a higher correlation coefficient (R^2^ = 0.83) and lower error rates (MAE = 14.64 and RMSE = 22.74) than other methods used in the prediction of *c_u_* value. Furthermore, the impact of input variables on the model output was investigated using the SHAP (SHApley Additive ExPlanations) technique based on the extreme gradient boosting (XGBoost) ensemble learning algorithm. The results demonstrated that using the GMDH-type NN is an efficient method in obtaining a new empirical mathematical model to provide a reliable prediction of the undrained shear strength of soils.

## 1. Introduction

Determining the engineering properties of soil layers is critical in geotechnical projects. Thus, field and laboratory tests are required in the evaluation of soil properties in geotechnical engineering. Field and laboratory experiments are the best ways to understand the complex behavior of soil layers, but those experiments involve several shortcomings. Among them, time and budget constraints are specially considered major disadvantages of the experiments. Geotechnical engineers need to determine critical soil properties without time and budget restrictions. However, many conventional field tests do not allow measuring required soil-design parameters directly; empirical correlations are commonly used to determine these design parameters [1].

The undrained shear strength (*c_u_*) is generally used as a geotechnical design parameter for clay soil, and it is one of these design parameters that is challenging to directly measure in the field. The *c_u_* value can be determined using unconfined or triaxial compression tests in a laboratory or using a hand penetrometer in the field. Estimating *c_u_* and unconfined compressive strength (*q_u_* = *c_u_*/2) is possible using the findings of the standard penetration test (SPT) performed in the field. The *q_u_* value is determined through unconfined compression or unconsolidated undrained (UU) tests. Many researchers have proposed various correlations between *N_SPT_* and *q_u_*. The correlation recommended by Terzaghi and Peck [2], between *q_u_* and *N_SPT_* in fine-grained soils is given in Table 1.

In geotechnical engineering, the SPT stands out as one of the most widely used methods in the field to evaluate the resistance of soil layers. The standard penetration resistance of soil layers is expressed by the number of blows, SPT N-value (*N_SPT_*), recorded for the last two 150-mm layers [3]. Here, the *N_SPT_* value does not directly provide the *c_u_* value that is used as a geotechnical design parameter. This situation has led many researchers to develop other correlations to predict the *c_u_* value using *N_SPT_* [4,5,6]. Table 2 shows the correlation between *N_SPT_* and *c_u_* according to soil classification from the literature [7,8,9].

Machine learning techniques have newly acquired great consideration among researchers in multiple disciplines [10]. Researchers have tried to develop the precise prediction and interpretability of models by using various decision-trees machine learning algorithms such as decision trees [11], random forests [12], classification and regression trees [13], and support vector regression [14]. Also, numerous research groups around the world have recognized the extraordinary potential these algorithms can bring to geotechnical engineering. Some researchers used a number of machine learning approaches (i.e., artificial neural network (ANN), support vector regression (SVR), and random forest (RF)) to predict cone penetration test (CPT) data according to soil classification. The results clearly indicated that both ANN and RF techniques showed precise predictions [15]. Some other researchers utilized machine learning techniques to estimate the stability of organic soils and they demonstrated that the ANN models showed the best precision accuracy [16,17]. Choi et al. [18] showed a few learning algorithms (deep neural networks, RF, and SVR) to estimate the leakage stress that is employed during drilling in the petroleum industry. Genetic algorithms were utilized to optimize site surveys for the design of pile foundations [19]. In addition, the group method of data handling (GMDH-type NN) approach was used for several geotechnical engineering applications. Mola-Abasi and Eslami [20], derived the GMDH models to predict shear strength parameters (*c* and *φ*) from CPTu data. Choobbasti and Valizadeh [21], used the GMDH-type NN to determine the optimal amount of clay and Nano-CuO to obtain the maximum undrained cohesion. In particular, Kalantary et al. [6] developed a mathematical model using an optimized GMDH-NN with a genetic algorithm and designed a correlation between *c_u_* and *N*_60_. Also, Mbarak et al. [1] examined the relationship between parameters obtained by the undrained shear strength (*c_u_*) and SPT test in fine-grained soils with their statistical model based on soil physical properties.

This study presents mathematical models that predict *c_u_* values using artificial intelligence technology. Additionally, the objective of this study is to obtain the most effective model for the prediction of undrained shear strength. The GMDH-type NN was compared with other models (i.e., SVR and RF) and classical regression (i.e., linear regression); the performance of the models was determined by using the correlation coefficient (R^2^), root-mean-square error (RMSE), and mean absolute error (MAE). In the prediction of the *c_u_* value, various soil parameters were used as input parameters and the models were made by various soil types. Moreover, the effect of the input variables on the prediction model was evaluated with the SHAP (SHApley Additive ExPlanations) approach according to the extreme gradient boosting (XGBoost) ensemble learning algorithm in this study. The novelty and main contributions of the study are as follows:

- Prediction of undrained shear strength was provided with high accuracy by the polynomial neural network based on the GMDH-type NN approach;
- SPT-value and soil physical properties input variables were analyzed with the XGBoost ensemble learning-based SHAP approach;
- It was ensured that the prediction model was obtained only with high-impact inputs;
- As an alternative to traditional methods, it is provided to obtain a self-organized predictive model for undrained shear strength;
- A predictive model with high performance on a small dataset was designed and implemented.

## 2. Methods and Procedures

### 2.1. Dataset

According to the findings of the literature [22,23,24], a total of 211 samples from soils classified as CH, CL, MH, and ML were used to assemble the dataset. This study was conducted using the results of *N_SPT_*, effective stress (*σ_v_*’), *c_u_*, *PL, LL*, and *PI* of the 211 samples.

**Figure 1:**
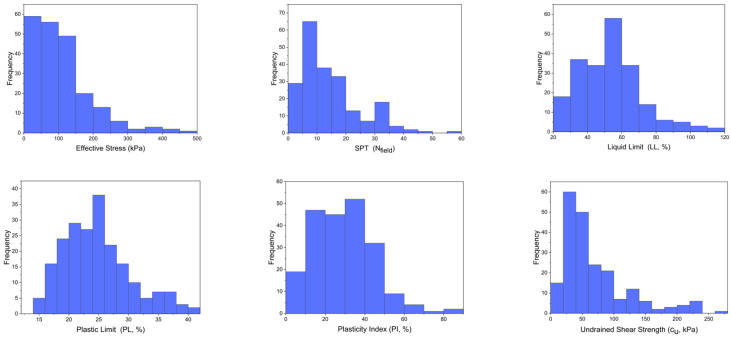
Histogram of the variables used for the model development.

Figure 1 presents the data with frequency histograms to reflect the distribution of the dataset. In addition, it shows that the statistical assumptions were met with the provision of multivariate normal distribution of the data, nonuniform distribution of independent (predictor) variables, non-linearity among independent (predictor) variables, and covariance. In addition, Table 3 presents the data as the descriptive statistics of the variables.

### 2.2. Pre-Processing

In order to build up a decent and widely applicable prediction model, the independent variables must be normalized and identified within a certain range. A min–max method is frequently used in the normalization of variables. The min–max method normalizes the independent variables of the data to the [0,1] range. The normalization of the min–max method is expressed as follows:

(1) $$x_{norm}=\frac{x-\min\left(x\right)}{\max\left(x\right)-\min\left(x\right)}$$

where *x* and *x_norm_* represent data value and normalized data, respectively.

### 2.3. Machine Learning Approaches

ANN models, such as multilayer perception, are extensively used in regression and classification. A multilayered neural network comprises one input, multiple hidden, and one output layer. In this network model, each cell in the hidden layer uses all inputs in the input layer. The use of all inputs in all cells in the network structure may cause overfitting problems and reduce performance. Difficulties and deficiencies are encountered in setting bias and weight coefficients, especially when handling small-sized data sets.

**Figure 2:**
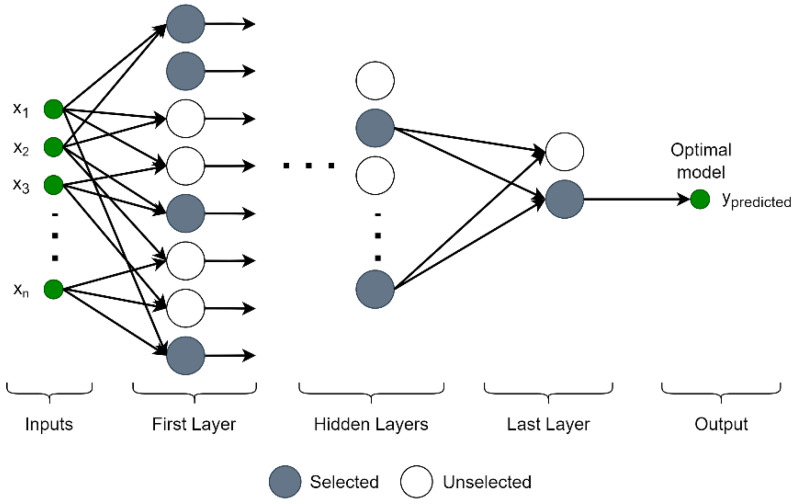
The schematic representation of GMDH-type NN.

Therefore, instead of this network model in which all inputs and cells in all layers are used, the GMDH-type NN, a self-organizing network model that acts based on the input data, is preferred [25,26]. The schematic structure of the GMDH-type NN is shown in Figure 2.

The GMDH-type NN is one of the best model prediction methods for problems involving complex structures. The GMDH-type NN model is a multilayered structure using only the cells that can yield the most efficient and accurate results. Each layer comprises independent cells used in pairs and is integrated with a quadratic polynomial as the activation function. The cells in all layers run independently from each other, and only the outputs from the previous layer that minimize the error rate are preferred. Thus, instead of using cells in all layers, the best network model comprising optimal cells is created [27]. The GMDH-type NN is used as a model that maps a given input vector $X=\left(x_{1},x_{2},\cdots ,x_{n}\right)$ to the predicted ${\bar{y}}_{i}$ output. It is expected that the predicted ${\bar{y}}_{i}$ output is as close as possible to the actual $y_{i}$ output. Thus, *M* results obtained for data pairs in a multi-input single-output network model are observed as follows [28]

(2) $$y_{i}=f\left(x_{i1},x_{i2},\cdots ,x_{in}\right)\;\;i=1,\;2,\;\ldots ,M$$

The output predicted to obtain ${\bar{y}}_{i}$ output from input vector $X=\left(x_{i1},x_{i2},\cdots ,x_{in}\right)$ is shown as follows:

(3) $${\bar{y}}_{i}=\bar{f}\left(x_{i1},x_{i2},\cdots ,x_{in}\right)\;\;i=1,\;2,\cdots ,M$$

The least-squares method is applied between the actual outputs $y_{i}$ and predicted outputs ${\bar{y}}_{i}$ to determine the GMDH model. The cells in which the errors calculated using the least-squares method are minimized are selected:

(4) $$\sum_{i=1}^{M}{\left(\bar{f}\left(x_{i1},x_{i2},\cdots ,x_{in}\right)-y_{i}\right)}^{2}\to minimum$$

The GMDH-type NN is identified based on input and output parameters emphasized in the form of the gradually complicated Kolmogorov–Gabor polynomial function [29]. Expressed as a nonlinear function form, the Kolmogorov–Gabor function is defined as:

(5) $$\bar{y}=\alpha_{0}+\sum_{i=0}^{n}\alpha_{i}x_{i}+\sum_{i=1}^{n}\sum_{j=1}^{n}\alpha_{ij}x_{i}x_{j}+\sum_{i=1}^{n}\sum_{j=1}^{n}\sum_{k=1}^{n}\alpha_{ijk}x_{i}x_{j}x_{k}+\cdots$$

Here, *α* shows the polynomial coefficients and (i, j, k)∈(1, 2,⋯,n). Typically, the Kolmogorov–Gabor polynomial, which gives a nonlinear polynomial form, is written in the form of a quadratic polynomial containing only two variables [27]:

(6) $$\bar{y}=G\left(x_{i},\;x_{j}\right)=\alpha_{0}+\alpha_{1}x_{i}+\alpha_{2}x_{j}+\alpha_{3}x_{i}x_{j}+\alpha_{4}x_{i}^{2}+\alpha_{5}x_{j}^{2}$$

The GMDH-type NN predicts the output for each set of input parameters $x_{i}$ and $x_{j}$ and is used to predict *α_i_* (1, 2, …, 5) coefficients that reduce RMSE between the estimated and real outputs. With *M* representing the total number of data, minimizing the RMSE between the predicted and actual outputs is as follows:

(7) $$E=\frac{\sum_{i=1}^{M}{\left({\bar{y}}_{i}-y_{i}\right)}^{2}}{M}\to minimum$$

In the basic form of the GMDH algorithm, all binary probabilities of independent variables from *n* inputs in total provide the establishment of the regression structure using the polynomial form given in Equation (6) to obtain the actual output data $\left(y_{i},\;i=1,\;2,\cdots ,M\right)$. The cell count in the hidden layer in the GMDH network model structure is determined by $\left(\begin{array}{c} n\\ 2 \end{array}\right)=n\left(n-1\right)/2$. In the next step, creating *M* data triples as $\left(y_{i},\;x_{ip},\;x_{iq}\right)$ (p,q)∈(1, 2,⋯,n) from the actual output data is possible. The resulting matrix form can be expressed as:

(8) $$\left[\begin{array}{cccc} x_{1p} & x_{1q} & \vdots & y_{1}\\ x_{2p} & x_{2q} & \vdots & y_{2}\\ \cdots & \cdots & \cdots & \cdots \\ x_{Mp} & x_{Mq} & \vdots & y_{M} \end{array}\right]$$

The essential form of the GMDH algorithm is expressed in matrix form and Equation (5) can be rewritten as:

(9) Y=Aα

Here $Y={\left\{y_{1},\;y_{2},\cdots ,y_{M}\right\}}^{T}$ represents the actual output vector, and $\alpha =\left\{\alpha_{1},\alpha_{2},\cdots ,\alpha_{5}\right\}$ presents the unknown coefficient vector of the quadratic polynomial vector. The predicted *A* matrix is expressed for different *p*, *q* as follows:

(10) $$A=\left[\begin{array}{cccccc} 1 & x_{1p} & x_{1q} & x_{1p}x_{1q} & x_{1p}^{2} & x_{1q}^{2}\\ 1 & x_{2p} & x_{2q} & x_{2p}x_{2q} & x_{2p}^{2} & x_{2q}^{2}\\ \cdots & \cdots & \cdots & \cdots & \cdots & \cdots \\ 1 & x_{Mp} & x_{Mq} & x_{Mp}x_{Mq} & x_{Mp}^{2} & x_{Mq}^{2} \end{array}\right]$$

The least-squares method for multi-regression analysis solves the normal equation as follows:

(11) $$\alpha ={\left(A^{T}A\right)}^{-1}A^{T}Y$$

The resultant *α* coefficients give the best coefficient vector of the quadratic polynomial given in Equation (6) for all *M* data triangulation. After calculating the descriptive coefficient vector of the quadratic polynomial, the objective function is used as a selection criterion to eliminate the cells with high error rates.

(12) $$OF=\frac{1}{n}\sum_{i=1}^{n}{\left(y_{pre}-y_{mea}\right)}^{2}$$

Here, *y_pre_* and *y_mea_* are the predicted and actual outputs, respectively; *n* indicates the total number of data. 

The SVR method separates the dataset with the help of a hyperplane and minimizes the errors within the boundary line. A kernel function is used to perform linear separation in the dataset. The SVR method, which creates an optimal hyperplane between data points, provides curve fitting with the maximum number of data [30,31]. In the prediction model obtained by training the data, linear, radial basis, and polynomial kernel functions can be used. The main benefit of SVR is that computational complexity is independent of input space size [32]. Additionally, it has a strong capacity for generalization and good prediction accuracy. The SVR, a method of supervised learning trains with a loss function that penalizes both high and low erroneous predictions equally.

The RF regression method is a machine learning method that can predict accurately in predictive analysis when the target output parameter and input variables have a non-linear relationship [33,34]. The RF technique is a supervised learning algorithm that does regression using the ensemble learning approach. The ensemble learning method combines predictions from different machine learning algorithms to produce a more accurate forecast from a single model. The predictions of all decision trees are combined to provide more accurate outputs in the RF algorithm, which reduces overfitting in model training. The variety of trees used increases the robustness of the model obtained as a result of regression [35]. RF regression models generally show strong and accurate performance on parameters with nonlinear relationships. The disadvantages can be listed as the lack of interpretability, the occurrence of over-fitting, and the need to select the number of trees included in the model.

The linear regression method, which is one of the simplest regression methods that provide the prediction of a parameter, is frequently preferred due to its straightforward and useful mathematical structure [36]. In the linear regression method, the mathematical equation of the target parameter to be predicted is obtained using a slope and intercept value. The target parameter and the input variables are shown by linear regression as:

(13) $$Y=a_{0}+a_{1}x_{1}+a_{2}x_{2}+\cdots +a_{n}x_{n}$$

where Y denotes output, $x_{1},x_{2},\cdots ,x_{n}$ and $a_{1},a_{2},\cdots ,a_{n}$ represent input variables and the coefficients of the regression model, respectively.

### 2.4. Performance Evaluation Metrics

Various metrics can be used to measure and evaluate the performance of prediction models. In this study, the R^2^, RMSE, and MAE values of the predicted and actual target parameters are calculated in the performance evaluation of regression models. The R^2^, RMSE, and MAE are expressed mathematically as:

(14) $$R^{2}=1-\frac{\sum_{i=1}^{N}{\left(y_{mea}-y_{pre}\right)}^{2}}{\sum_{i=1}^{N}{\left(y_{mea}-y_{m}\right)}^{2}}$$

(15) $$\mathrm{RMSE}=\sqrt{\frac{\sum_{i=1}^{N}{\left(y_{mea}-y_{pre}\right)}^{2}}{N}}$$

(16) $$\mathrm{MAE}=\frac{1}{N}\sum_{i=1}^{N}\left|y_{mea}-y_{pre}\right|$$

where *y_mea_*, *y_pre_*, and *y_m_* denote the average of actual output, estimated output, and actual output, respectively. *N* represents the total number of data. The degree of fitting increases with R^2^ proximity to 1. RMSE and MAE are used to evaluate the model’s prediction ability. For the RMSE and MAE, the prediction model will be more accurate and its accuracy will be higher with a smaller value.

### 2.5. Procedures

**Figure 3:**
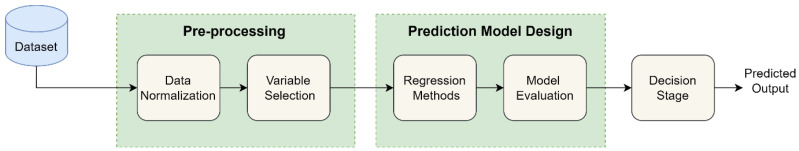
Block diagram of the proposed approach for prediction of undrained shear strength.

The proposed approach for the prediction of *c_u_* is given in Figure 3. In order to predict *c_u_*, the dataset is first put into the pre-processing phase. In this phase, the data should be initially normalized and independent variables should be selected. Then, a prediction model design phase is started. In this phase, different regression methods are applied and performance evaluation is calculated for different regression methods.

The GMDH-type NN approach is used to obtain the models and the results are compared with the most commonly used linear, RF, and SVR methods. Tree numbers [50,100,200,500] were selected for the RF model and the optimal number of trees was set to 50 by parameter grid search. The radial basis, which provides the best performance, was used as the core function for the SVR model. In addition, the degree and penalty parameter C were set to 3 and 1, respectively.

In the GMDH-type NN algorithm, the maximum number of layers and neurons in each layer is set to 5 as initial values. Then, the optimal number of layers and neurons was determined to minimize the error between the target and the predicted output using a grid search algorithm. Also, the selection pressure value on the layers is set to 0.8. In the design of the prediction models, 80% of the dataset is used for training and 20% for testing.

**Figure 4:**
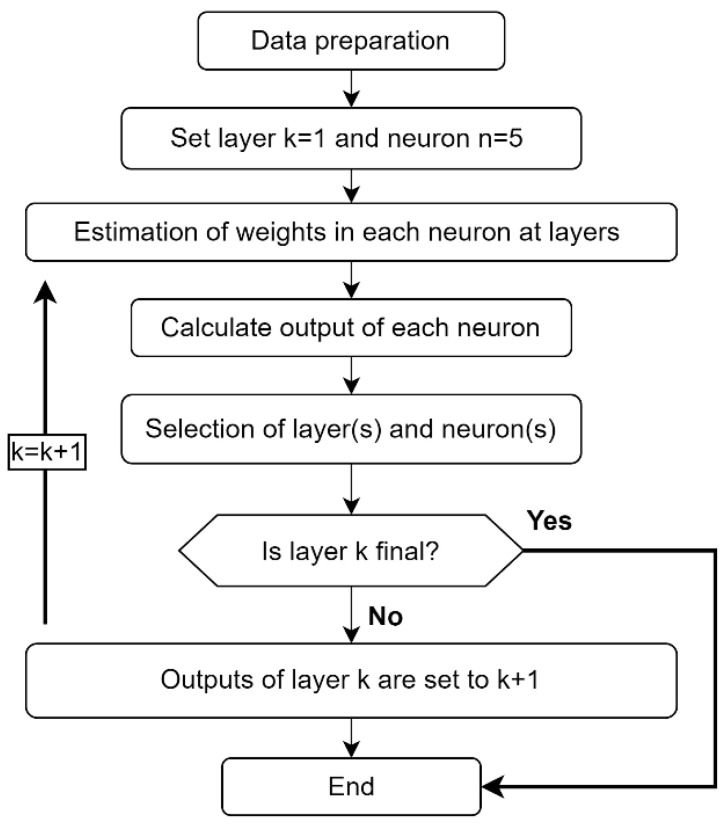
Flowchart of the proposed GMDH-type NN approach.

In this study, train and test data were randomly selected and the performance evaluation of the produced prediction models was obtained as a result of 10 trials. Figure 4 shows the flowchart of the proposed GMDH-type NN approach.

The GMDH-type NN, which is a machine learning-based method, is preferred to obtain a new and effective mathematical model that can be used to predict undrained shear strength. The GMDH-type NN structure, wherein input parameters are used as a binary polynomial, selects the most suitable neurons that minimize errors, and predicts output shear strength values accurately and effectively. Four different GMDH-type NN models are designed for various input parameters to obtain mathematical models for the *c_u_* prediction. The R^2^, MAE, and RMSE values are obtained for all prediction models designed and compared with the regression models (i.e., linear, RF, and SVR).

## 3. Results and Discussion

**Figure 5:**
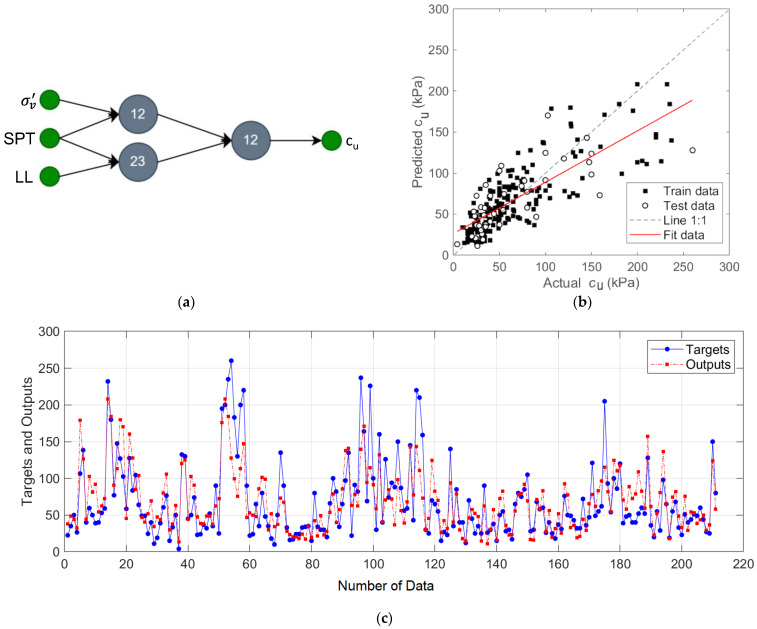
The proposed prediction model for input variables *σ_v_*’, *N_SPT_*, and *LL*: (**a**) two-layers GMDH-type NN structure; (**b**) regression plot; (**c**) target and output predictive values.

In the first designed GMDH-type NN model, the prediction of *c_u_* was performed for the input variables *σ_v_*’, *N_SPT_*, and *LL*. The network structure, regression plot, and output prediction values for this model are shown in Figure 5.

In this network structure shown in Figure 5a, the pairs of input variables {*σ_v_*’ and *N_SPT_*} and {*N_SPT_* and *LL*} were processed in two neurons, and the outputs of these neurons were formed into a pair and the predicted output was obtained. The results showed that this two-layer model has R^2^ of 0.79, MAE of 6.02, and RMSE of 24.94 values.

**Figure 6:**
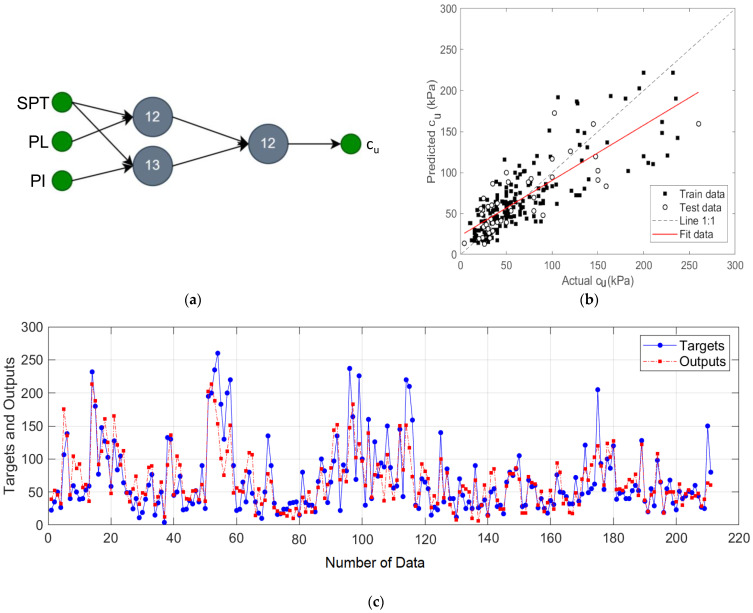
The proposed prediction model for input variables *N_SPT_*, *PL*, and *PI*: (**a**) two-layers GMDH-type NN structure; (**b**) regression plot; (**c**) target and output predictive values.

The GMDH-type NN model designed with *N_SPT_*, *PL*, and *PI* input variables is shown in Figure 6a and the results of this model are given in Figure 6b,c. In this designed model, input variable pairs {*N_SPT_* and *PL*} and {*N_SPT_* and *PI*} were processed in two neurons, and prediction of output was performed by creating a pair of these neuron outputs. In this model, where the best results were obtained with two layers; NN, R^2^ of 0.82, MAE of 14.65, and RMSE of 23.05 values were achieved. Figure 6c shows the target and predicted output values for this model.

**Figure 7:**
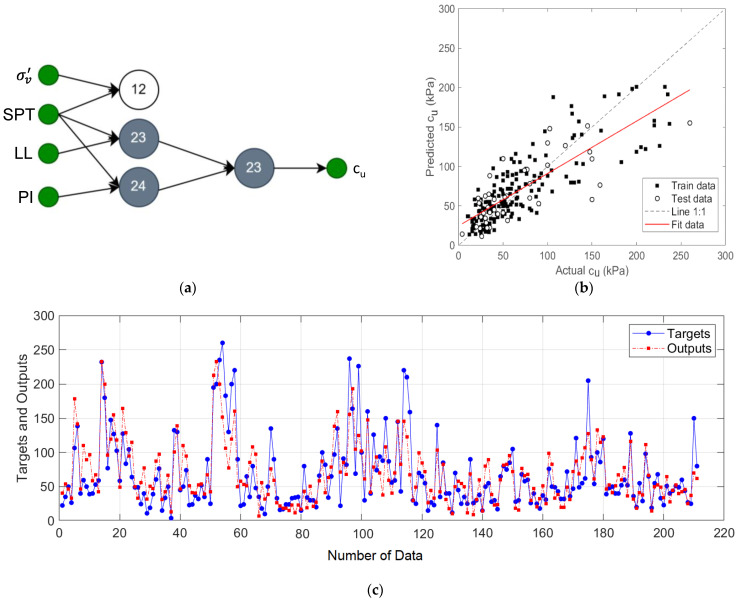
The proposed prediction model for input variables *σ_v_*’, *N_SPT_*, *LL*, and *PL*: (**a**) two-layer GMDH-type NN structure; (**b**) regression plot; (**c**) target and output predictive values.

The GMDH-type NN structure for the model was designed using *σ_v_*’, *N_SPT_*, *LL*, and *PI* input variables, and the results for this model are given in Figure 7. In the first layer of this network, neuron outputs for {*σ_v_*’, *N_SPT_*}, {*N_SPT_*, *LL*} and {*N_SPT_*, *PI*} input pairs were calculated and {*σ_v_*’, *N_SPT_*} output from these neurons was disabled because it increased the error rate. Then, the remaining neuron outputs are paired to generate the predicted output. The R^2^, MAE, and RMSE of this designed model are 0.82, 14.91, and 22.88, respectively.

**Figure 8:**
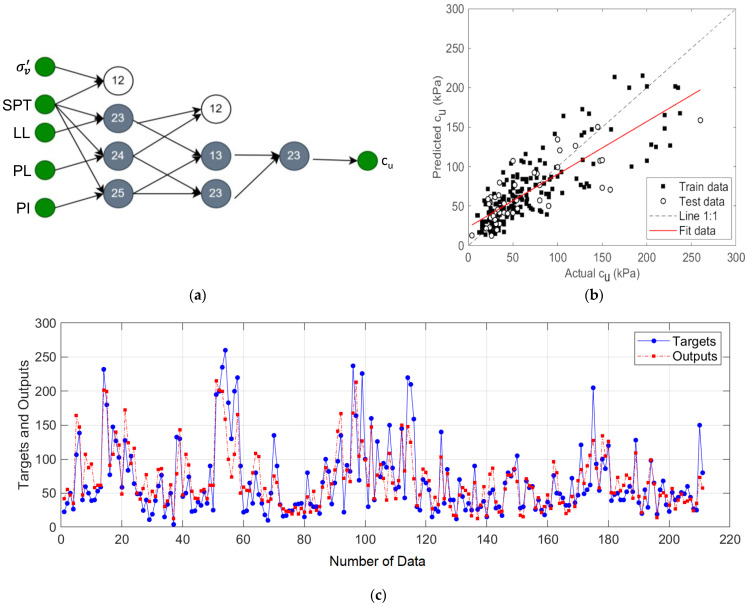
The proposed prediction model for input variables *σ_v_*’, *N_SPT_*, *LL, PL*, and *PI*: (**a**) three-layer GMDH-type NN structure; (**b**) regression plot; (**c**) target and output predictive values.

The GMDH-type NN structure using all input variables and the performance results of this model are given in Figure 8. In this model, the variable pair {*σ_v_*’, *N_SPT_*} was disabled because it increased the error rate, and the remaining neuron outputs formed pairs and were used in the next layer. The results given in Figure 8b,c show that the GMDH-type NN model achieved R^2^ of 0.83, MAE of 14.64, and RMSE of 22.74 values.

In order to evaluate the performance of prediction models, the GMDH-type NN method was compared with linear regression, RF, and SVR methods. Table 4 summarizes the performance evaluation of linear, RF, SVR, and GMDH-type NN regression models with different input parameters in the prediction of *c_u_*.

As mentioned above, the four regression methods were used to design the prediction models of *c_u_*. The results of R^2^ on average for the linear, RF, SVR, and GMDH, which imply a higher degree of fitting, are 0.5 ± 0.04, 0.55 ± 0.02, 0.61 ± 0.02, and 0.82 ± 0.02, respectively. In addition, the results of MAE on average for the four prediction models are 23.68 ± 0.75, 20.95 ± 0.33, 18.38 ± 0.87, and 15.06 ± 0.57, respectively. Also, the RMSE values are 33 ± 1.28, 31.06 ± 0.83, 28.98 ± 0.58, and 23.4 ± 0.9 corresponding to the linear, RF, SVR, and GMDH methods. The linear, RF, and SVR have R^2^ values of about 0.5, while the GMDH-type NN method had the highest R^2^ values of 0.8. 

The linear, RF, and SVR methods show that the R^2^ value is independent of the number of input parameters. In the linear regression and SVR methods, the highest R^2^ value and lowest error rates have been achieved when the input parameters were *σ_v_*’, *N_SPT_* and *LL*. In addition, when the input parameters were *N_SPT_*, *PL*, and *PI*, the highest R^2^ value and lowest error rates have been obtained in the RF method. However, when increased the number of input parameters (i.e., *σ_v_*’, *N_SPT_*, *LL, PL*, and *PI*), a higher R^2^ value is achieved in the GMDH-type NN. The highest prediction performance was obtained with the GMDH-type NN by using *σ_v_*’, *N_SPT_*, *LL, PL*, and *PI* input variables. This proposed model had the highest R^2^ value and the lowest MAE and RMSE values. As a result of the evaluation, the GMDH-type NN approach shows more reliability in estimating *c_u_* than other regression methods.

**Figure 9:**
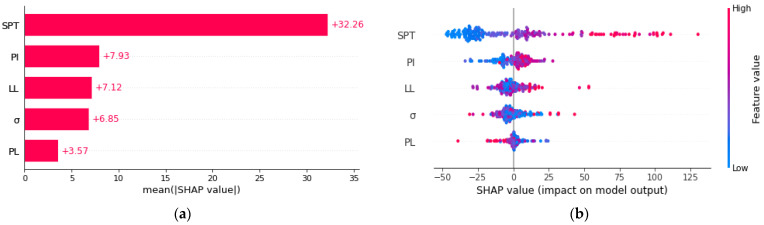
The variable importance for prediction models: (**a**) the average SHAP value of each input variable; (**b**) the sum of the SHAP value magnitudes of the input variables on all samples.

The effect of the input variables on the model output was additionally observed with the SHAP (SHApley Additive ExPlanations) approach. The SHAP is a game-theoretic method for expressing any machine learning model’s output. The SHAP determines the ideal parameters by using the conventional Shapley values from game theory and their related extensions. Extreme gradient boosting (XGBoost) decision tree, which is a high-speed exact algorithm, was used to obtain SHAP values. Figure 9 shows the average SHAP values of the input variables on the model output.

Figure 9a shows the average SHAP value of each input variable. Average SHAP results clearly indicate that the input variable of *N_SPT_* has the most effect on the *c_u_* prediction compared to other input variables. The summation of the SHAP value magnitudes of the input variables on all samples and the sorting and distribution of the effects of each variable on the model output is shown in Figure 9b. The results reveal that the *N_SPT_* value significantly affects the prediction of *c_u_* compared to other input parameters.

## 4. Conclusions

This study investigated the performance of machine learning models for the prediction of *c_u_*. The GMDH-type NN approach was used with varying input parameters in order to design the prediction models. In the *c_u_* prediction models, *N_SPT_*, *σ_v_*’, *LL, PL*, and *PI* were used as input variables. Then, the results of GMDH-type NN were compared with the most commonly used linear regression, RF, and SVR methods. The performance of those methods was evaluated with the R^2^, MAE, and RMSE. Also, the effect of the number of input parameters for prediction models was studied. In addition, since the four prediction models for each method have been derived with different input variables, the most effective input parameter was determined by using a game-theoretic approach.

The results showed that the proposed a three-layer GMDH-type NN model had a higher regression coefficient (R^2^) and lower error rates (MAE and RMSE) in all combinations of input parameters compared to other methods used in the prediction of *c_u_*. Moreover, the results revealed that when we increased the number of input parameters (i.e., *σ_v_*’, *N_SPT_*, *LL, PL*, and *PI*) in the GMDH-type NN model, the model achieved high reliability compared to the lower number of input parameters.

The effects of the input variables on the prediction model were evaluated with the SHAP (SHApley Additive ExPlanations) approach based on the extreme gradient boosting (XGBoost) ensemble learning algorithm. The SHAP results revealed that the *N_SPT_* value was the most influential parameter in the estimation of the *c_u_* value compared to other parameters for all prediction models.

In summary, the results of the GMDH-type NN model showed that it is feasible to use artificial intelligence technology for the prediction of the undrained shear strength (*c_u_*). By using the GMDH-type NN approach, the problems of experimental restrictions can be solved, and more accurate values can be achieved in the prediction of *c_u_*. Moreover, this method is expected to be well used to predict other geotechnical design parameters and further understand the complex behavior of clay soil.

## Figures and Tables

**Table 1:** The correlation between *q_u_* and *N_SPT_* [2].

Consistency	*N_SPT_*	*q_u_* (kPa)
Very soft	<2	25
Soft	2–4	25–50
Medium	4–8	50–100
Stiff	8–15	100–200
Very stiff	15–30	200–400
Hard	>30	>400

**Table 2:** Correlations between *N_SPT_* and *c_u_* based on soil classification.

Reference	Soil Classification	*c_u_* (kPa)
Stroud [7]	*PI* < 20	6–7 *N_SPT_*
	20 < *PI* < 30	4–5 *N_SPT_*
	*PI* > 30	4.2 *N_SPT_*
Dècourt [8]	Clay	12.5 *N_SPT_*
	Clay	15 *N_60_*
Sivrikaya ve Toğrol [9]	Low Plastic Clay	3.97 *N_field_*
	Low Plastic Clay	5.82 *N_60_*
	High Plastic Clay	5.90 *N_field_*
	High Plastic Clay	8.76 *N_60_*
	Clay	5.13 *N_field_*
	Clay	7.57 *N_60_*
	Fine-grained soil	4.68 *N_field_*
	Fine-grained soil	6.97 *N_60_*

**Table 3:** Descriptive statistics of parameters.

Variable	Minimum	Maximum	Mean	Standard Deviation
Effective stress (*σ_v_*’, kPa)	15	486	108.98	84.77
SPT N-value (*N_SPT_*, blows)	2	57	13.96	10.17
Liquid limit (*LL*, %)	24	118	52.74	17.82
Plastic limit (*PL*, %)	14	40	24.18	5.61
Plasticity index (*PI*, %)	2	84	28.63	15.14
Undrained shear strength (*c_u_*, kPa)	4	260	68.19	52.53

**Table 4:** Comparative results of the *c_u_* prediction models for different input variables.

Input Parameter	Regression Methods											
	Linear			RF			SVR			GMDH		
	R^2^	MAE	RMSE	R^2^	MAE	RMSE	R^2^	MAE	RMSE	R^2^	MAE	RMSE
$\sigma_{v}^{’}$, *N_SPT_*, *LL*	0.56	22.38	30.78	0.52	21.34	32.23	0.63	17.30	28.13	0.79	16.02	24.94
*N_SPT_*, *PL, PI*	0.48	24.06	33.62	0.58	20.92	29.93	0.60	19.22	29.48	0.82	14.65	23.04
$\sigma_{v}^{’}$, *N_SPT_*, *LL, PI*	0.47	24.17	33.76	0.56	20.45	30.79	0.62	17.76	28.77	0.82	14.91	22.88
$\sigma_{v}^{’}$, *N_SPT_*, *LL, PL, PI*	0.47	24.09	33.84	0.55	21.10	31.28	0.59	19.24	29.55	0.83	14.64	22.74

## References

[1] Mbarak W.K., Cinicioglu E.N., Cinicioglu O. (2020). SPT based determination of undrained shear strength: Regression models and machine learning. Front. Struct. Civ. Eng..

[2] Terzaghi K., Peck R.B. (1967). Soil Mechanics in Engineering Practice.

[3] Mohamed A. (2017). El-Reedy. Soil Investigation and Pile Design. Onshore Structural Design Calculations: Power Plant and Energy Processing Facilities.

[4] Hara A., Ohta T., Niwa M., Tanaka S., Banno T. (1974). Shear modulus and shear strength of cohesive soils. Soils Found..

[5] Sivrikaya O., Togrol E. (2006). Determination of undrained strength of fine grained soils by means of SPT and its application in Turkey. Eng. Geol..

[6] Kalantary F., Ardalan H., Nariman-Zadeh N. (2009). An investigation on the S_u_-N_SPT_ correlation using GMDH type neural networks and genetic algorithms. Eng. Geol..

[7] Stroud M.A. The standard penetration test in insensitive clays and soft rock. Proceedings of the European Symposium on Penetration Testing.

[8] Décourt L. (1990). The Standard Penetration Test: State-of-the-Art-Report.

[9] Sivrikaya O., Togrol E. (2007). Relationships between SPT-N value and undrained shear strength of fine-grained soils in Turkey. Tek. Dergi.

[10] Thai Pham B., Shirzadi A., Shahabi H., Omidvar E., Singh S.K., Sahana M., Telebpour Asl D., Bin Ahmad B., Kim Quoc N., Lee S. (2019). Landslide susceptibility assessment by novel hybrid machine learning algorithms. Sustainability.

[11] Tsangaratos P., Ilia I. (2016). Landslide susceptibility mapping using a modified decision tree classifier in the Xanthi Perfection, Greece. Landslides.

[12] Chen W., Xie X., Peng J., Shahabi H., Hong H., Bui D.T., Duan Z., Li S., Zhu A.-X. (2018). Gis-based landslide susceptibility evaluation using a novel hybrid integration approach of bivariate statistical based random forest method. Catena.

[13] Chen W., Shahabi H., Shirzadi A., Hong H., Akgun A., Tian Y., Liu J., Zhu A.X., Li S. (2019). Novel hybrid artificial intelligence approach of bivariate statistical-methods-based kernel logistic regression classifier for landslide susceptibility modeling. Bull. Int. Assoc. Eng. Geol..

[14] Tien Bui D., Shahabi H., Shirzadi A., Chapi K., Hoang N.D., Pham B., Bui Q.T., Tran C.T., Panahi M., Bin Ahamd B. (2018). A novel integrated approach of relevance vector machine optimized by imperialist competitive algorithm for spatial modeling of shallow landslides. Remote Sens..

[15] Rauter S., Tschuchnigg F. (2021). CPT Data Interpretation Employing Different Machine Learning Techniques. Geosciences.

[16] Hernandez-Martinez F., Al-Tabbaa A., Medina-Cetina Z., Yousefpour N. (2021). Stiffness and Strength of Stabilized Organic Soils—Part I/II: Experimental Database and Statistical Description for Machine Learning Modelling. Geosciences.

[17] Yousefpour N., Medina-Cetina Z., Hernandez-Martinez F., Al-Tabbaa A. (2021). Stiffness and Strength of Stabilized Organic Soils—Part II/II: Parametric Analysis and Modeling with Machine Learning. Geosciences.

[18] Choi J., Liu Z., Lacasse S., Skurtveit E. (2021). Leak-Off Pressure Using Weakly Correlated Geospatial Information and Machine Learning Algorithms. Geosciences.

[19] Crisp M., Jaksa M., Kuo Y. (2020). Optimal Testing Locations in Geotechnical Site Investigations through the Application of a Genetic Algorithm. Geosciences.

[20] Mola-Abasi H., Eslami A. (2019). Prediction of drained soil shear strength parameters of marine-deposit from CPTu data using GMDH-type neural network. Mar. Georesour. Geotechnol..

[21] Choobbasti A.J., Valizadeh M. (2022). The effect of nano-Cuo on mechanical, microstructural, and self-healing properties of clayey sandy soils. Arab. J. Geosci..

[22] Sivrikaya O. (2009). Comparison of Artificial Neural Networks models with correlative works on undrained shear strength. Eurasian Soil Sci..

[23] Sivrikaya O., Toğrol E., Bilgehan M. Estimating Undrained Shear Strength of Cohesive Soils by Means of Artificial Neural Networks. Proceedings of the 7th International Congress on Advances in Civil Engineering.

[24] Sivrikaya O. (2003). Determinatıon of Soil Properties by Means of Standard Penetration Test and Its Application in Turkey. Ph.D. Thesis.

[25] Kondo T. GMDH neural network algorithm using the heuristic self-organization method and its application to the pattern identification problem. Proceedings of the 37th SICE Annual Conference, International Session Papers.

[26] Anastasakis L., Mort N. (2001). The Development of Self-Organization Techniques in Modelling: A Review of the Group Method of Data Handling (GMDH).

[27] Elbaz K., Shen S.L., Zhou A., Yin Z.Y., Lyu H.M. (2021). Prediction of disc cutter life during shield tunneling with AI via the incorporation of a genetic algorithm into a GMDH-type neural network. Engineering.

[28] Oh S.K., Pedrycz W. (2002). The design of self-organizing polynomial neural networks. Inf. Sci..

[29] Farlow S.J. (2020). Self-Organizing Methods in Modeling: GMDH Type Algorithms.

[30] Smola A.J., Schölkopf B. (2004). A tutorial on support vector regression. Stat. Comput..

[31] Awad M., Khanna R. (2015). Support vector regression. Efficient Learning Machines.

[32] Sapankevych N.I., Sankar R. (2009). Time series prediction using support vector machines: A survey. IEEE Comput. Intell. Mag..

[33] Ahmad M.W., Jonathan R., Yacine R. (2018). Predictive modelling for solar thermal energy systems: A comparison of support vector regression, random forest, extra trees and regression trees. J. Clean. Prod..

[34] Salazar F., Toledo M.A., Oñate E., Morán R. (2015). An empirical comparison of machine learning techniques for dam behaviour modelling. Struct. Saf..

[35] Rodriguez-Galiano V., Sanchez-Castillo M., Chica-Olmo M., Chica-Rivas M. (2015). Machine learning predictive models for mineral prospectivity: An evaluation of neural networks, random forest, regression trees and support vector machines. Ore Geol. Rev..

[36] Montgomery D.C., Peck E.A., Vining G.G. (2012). Introduction to Linear Regression Analysis.

